# Detection for melanoma skin cancer through ACCF, BPPF, and CLF techniques with machine learning approach

**DOI:** 10.1186/s12859-023-05584-7

**Published:** 2023-12-06

**Authors:** P. Kavitha, G. Ayyappan, Prabhu Jayagopal, Sandeep Kumar Mathivanan, Saurav Mallik, Amal Al-Rasheed, Mohammed S. Alqahtani, Ben Othman Soufiene

**Affiliations:** 1 Department of Artificial Intelligence and Data Science, Panimalar Engineering College, Chennai, India; 2 Department of Information Technology, Prince Shri Venkateshwara Padmavathy Engineering College, Chennai, India; 3 School of Computer Science Engineering and Information Systems, Vellore Institute of Technology, Vellore, Tamil Nadu 632014 India; 4 School of Computing Science and Engineering, Galgotias University, Greater Noida, Uttar Pradesh 203201 India; 5 Department of Environmental Health, Harvard T H Chan School of Public Health, Boston, MA 02115 USA; 6 Department of Pharmacology and Toxicology, The University of Arizona, Tucson, AZ 85721 USA; 7 Department of Information Systems, College of Computer and Information Sciences, Princess Nourah bint Abdulrahman University, P.O. Box 84428, 11671 Riyadh, Saudi Arabia; 8 Radiological Sciences Department, College of Applied Medical Sciences, King Khalid University, 61421 Abha, Saudi Arabia; 9 BioImaging Unit, Space Research Centre, University of Leicester, Michael Atiyah Building, Leicester, LE1 7RH UK; 10 PRINCE Laboratory Research, ISITcom, Hammam Sousse, University of Sousse, Sousse, Tunisia

**Keywords:** Color layout filter, Auto color correlogram filter, Attribute selection classifier, Binary pattern pyramid filter, Bagging

## Abstract

Intense sun exposure is a major risk factor for the development of melanoma, an abnormal proliferation of skin cells. Yet, this more prevalent type of skin cancer can also develop in less-exposed areas, such as those that are shaded. Melanoma is the sixth most common type of skin cancer. In recent years, computer-based methods for imaging and analyzing biological systems have made considerable strides. This work investigates the use of advanced machine learning methods, specifically ensemble models with Auto Correlogram Methods, Binary Pyramid Pattern Filter, and Color Layout Filter, to enhance the detection accuracy of Melanoma skin cancer. These results suggest that the Color Layout Filter model of the Attribute Selection Classifier provides the best overall performance. Statistics for ROC, PRC, Kappa, F-Measure, and Matthews Correlation Coefficient were as follows: 90.96% accuracy, 0.91 precision, 0.91 recall, 0.95 ROC, 0.87 PRC, 0.87 Kappa, 0.91 F-Measure, and 0.82 Matthews Correlation Coefficient. In addition, its margins of error are the smallest. The research found that the Attribute Selection Classifier performed well when used in conjunction with the Color Layout Filter to improve image quality.

## Introduction

That much is certain: the skin is the body's largest organ. The body's critical organs are protected from the elements. The skin shields us from the sun's damaging rays, keeping our core body temperature consistent. [1, 2] It prevents damage from hazardous compounds and facilitates vitamin D production, both of which are essential to several bodily processes. Melanoma, a malignant tumour, most often develops and spreads in sun-exposed parts of the skin. However, this common malignancy can also develop in areas of the skin that see little sunlight. Globally, melanoma cases have been on the rise, causing a significant health burden. Melanoma ranks sixth among all skin cancers in terms of frequency of occurrence. Since it has the potential to metastasize to other parts of the body, the Skin Cancer Foundation (SCF) ranks it as the deadliest kind of skin cancer. Melanoma that has spread is nearly impossible to cure. Sometimes, early detection of rare but potentially lethal illnesses saves patients' lives. The most frequent type of skin cancer, melanomas, have been proven to return at regular intervals, and the incidence of skin cancer as a whole has risen steadily over the past few decades. Uncontrolled cell growth in the skin leads to skin disease, the most deadly form of cancer. Most skin cancers and diseases fall into one of three categories: melanomas, basal cell tumors, and squamous cell cancers [3]. Non-melanoma skin tumors are any malignant skin growths that aren't melanomas [4]. There are two distinct types of skin cancer: malignant and non-malignant. Squamous cell carcinoma and basal cell carcinoma are two examples of benign cancers. [5, 6] Intent-based malignant cancer is the most tragic and lethal kind of the illness. The most expensive types of cancer are those that affect the skin and the pores. This paper organizes the "Related works" section has related articles of related works; "Proposed technique" section has materials and methods; "Discussions" section has results and discussions and finally "Conclusion" section has conclusions of this research work. Melanoma, the deadliest kind of skin cancer that begins in melanocytes, is extremely common across the world, though [10–15] its incidence varies widely from region to region. Melanoma cases are documented worldwide, albeit they are more common in areas with high amounts of ultraviolet light like Australia and some portions of North America and Europe. Increased sun exposure, shifting behavioural patterns, and unknown environmental impacts have all been blamed for the worrying increase in melanoma prevalence over the past few decades. Due to its aggressive nature and tendency for metastasis, melanoma has a considerable impact on mortality despite its relatively modest share among skin cancer occurrences. Adults, especially men, have a higher risk of being diagnosed with the condition, though it can strike at any age. The prognosis for melanoma is strongly correlated with how early it is diagnosed. Fortunately, new immunotherapies and tailored medicines provide better options for care. Melanoma is preventable and treatable, but only if people are made aware of the risk and encouraged to take preventative actions on a global scale.

Significance of the study, the authors of this work have used the Auto Correlogram Methods, the Binary Pyramid Patter Filter, and the Ensemble Model to increase the accuracy of their detection of Melanoma skin cancer. The Color Layout Filter model of the Attribute Selection Classifier provides the best overall performance, with statistics for ROC, PRC, Kappa, F-Measure, and Matthews Correlation Coefficient showing 90.96% accuracy, 0.91 precision, 0.91 recall, 0.95 ROC, 0.87 PRC, 0.87 Kappa, 0.91 F-Measure, and 0.82 Matthews Correlation Coefficient. The research also found that the Attribute Selection Classifier performed well when used in conjunction with the Color Layout Filter to improve image quality.

## Related works

Various AI methods, such as the construction of multi-layered structures of input and output training data [1–3], have been applied to tackle complex problems in the healthcare system. In the field of skin cancer detection, algorithms such as XYZ and ABC have shown promising results. This paper aims to build upon this existing body of work and explore additional techniques. It is now possible to use deep l9earning approaches to solve problems that traditional ANNs have trouble with [4–7]. ANNs have been used for a broad variety of tasks, including but not limited to the following: the classification of written texts, the construction of image processing-based recognition systems, and the analysis of enormous volumes of scientific data [8, 9].

One of the most important areas where ANNs have been put to use is in the field of diagnosing diseases [16–20]. To assess the efficacy of health informatics, ANNs were used to analyse biological data and an MRI scan image [21–25]. Many biomedical tasks, such as cancer diagnosis, have been completed with the aid of AI systems. There are several applications of AI algorithms in the medical field, including image segmentation, the development of diagnostic systems, the categorization of diseases, the prediction of diseases [26–32] through health informatics, and the detection of targeted anatomical regions. Use of deep learning algorithms in the field of biomedical health has produced encouraging outcomes [25–33]. Skin cancer detection software on the computer. They investigated a variety of dermatological datasets to test their research models. [34–56]. To identify brain cancers in the test data, the algorithm outperformed state-of-the-art methods, with 96% accuracy for CNN, 98.5% accuracy for VGG 16, and 98.14% accuracy for the Ensemble Model. The F1-score was 91.78%, 92.6%, and 91.29%, and the precision was 96%, 98.15%, and 98.41%. [57] The research offers further recommendations for achieving higher levels of efficiency, say, better than 0.90 efficiency on a scale from 0 to 1. [58]. Researchers from all around have zeroed in on skin cancer and come up with new ways to diagnose and predict the disease [59–64].

## Proposed technique

This proposed system was implemented by ISIC 2018. This work considers around 10,000 images. They are specified in below Table 1.

**Table 1:** Meta data of ISIC (International Skin Imaging Collaboration) dataset

S. No.	Name of the class	Description
1	nv	Melanocytic nevi
2	mel	Melanoma
3	bkl	Benign keratosis lesions
4	bcc	Basal cell carcinoma
5	akiec	Actinic keratoses
6	vasc	Vascular
7	df	Dermatofibroma

### Methods

The following techniques are applied in this research work.

1. Image Acquisition
2. Image preprocessing
3. Apply Auto Color Correlogram Filter, Binary Patterns Pyramid Filter, and Color Layout Filter by producing 34 attributes.
4. Relate for machine learning algorithms
  - Attribute selection is used to minimize the dimensionality of both training and test data before they are handed on to a classifier.
  - Bagging-Class is used to reduce variation in a classifier by bagging.
5. To get an optimal solution.

To produce a final result, these techniques have been implemented in one of the top and open supply programs, Weka3.9.5. This observation makes use of handiest 10% of the complete dataset and makes use of tenfold go validation for all categories.

### Experimental result

**Fig. 1:**
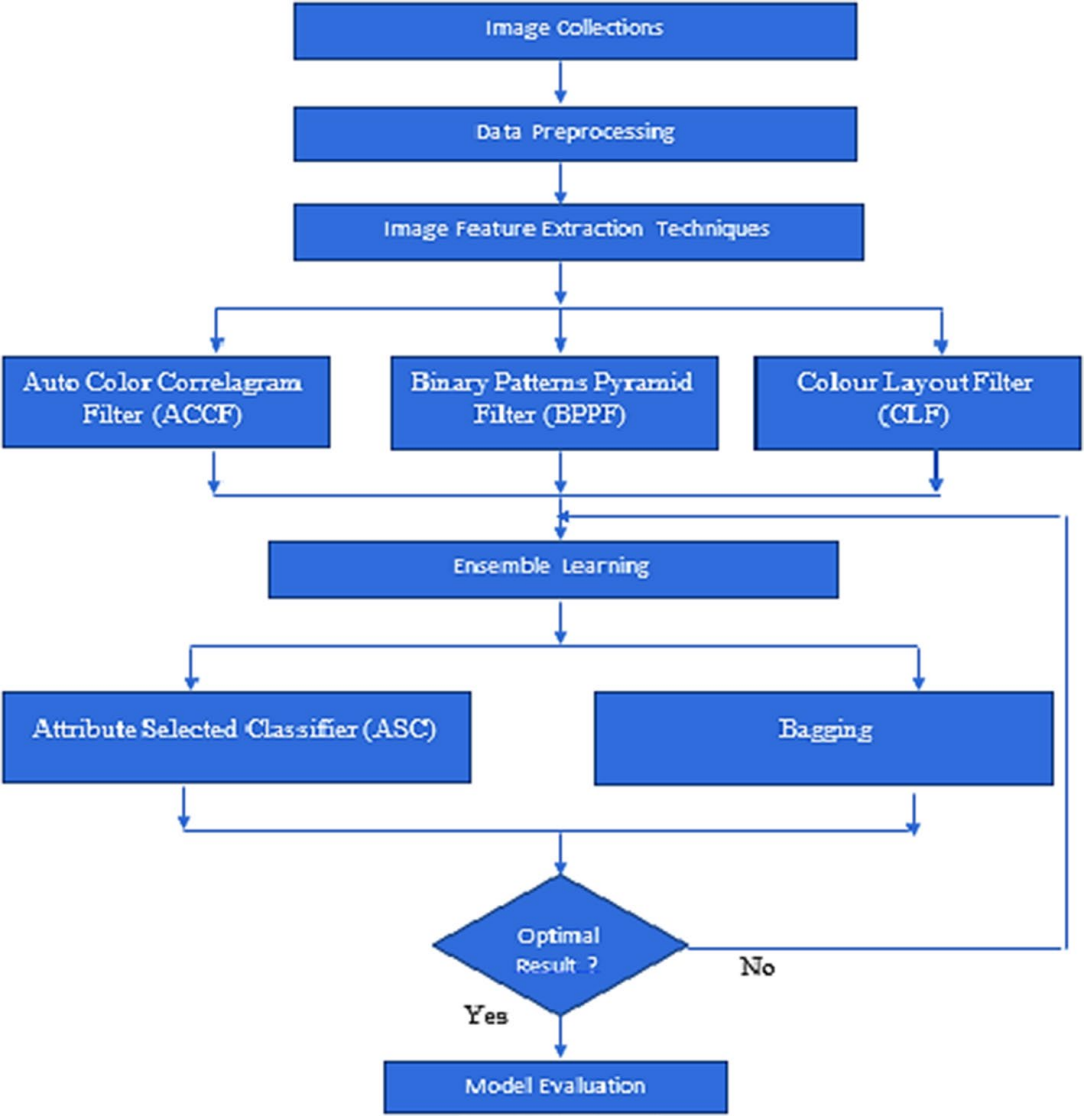
Methodology proposal

**Fig. 2:**
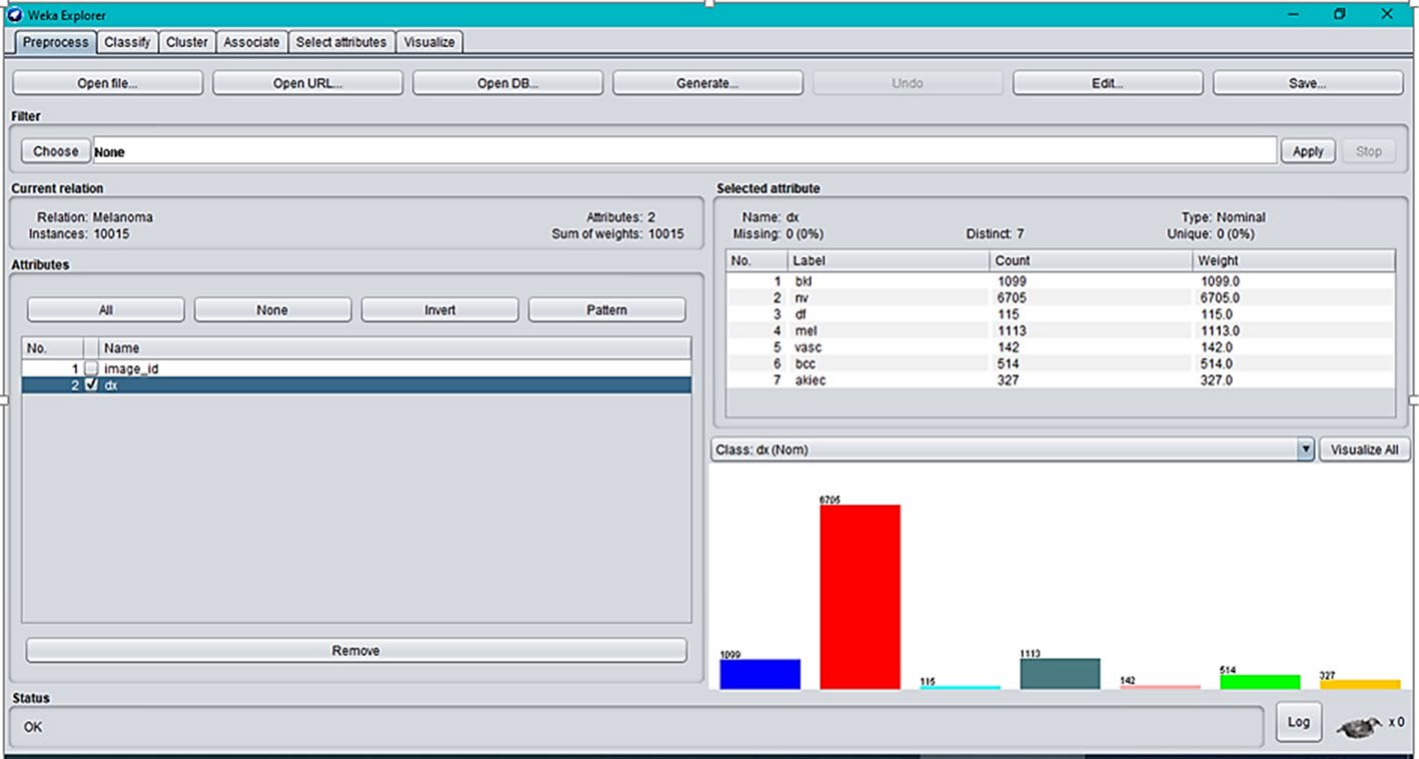
Representation of dataset in Weka.3.9.5

**Fig. 3:**
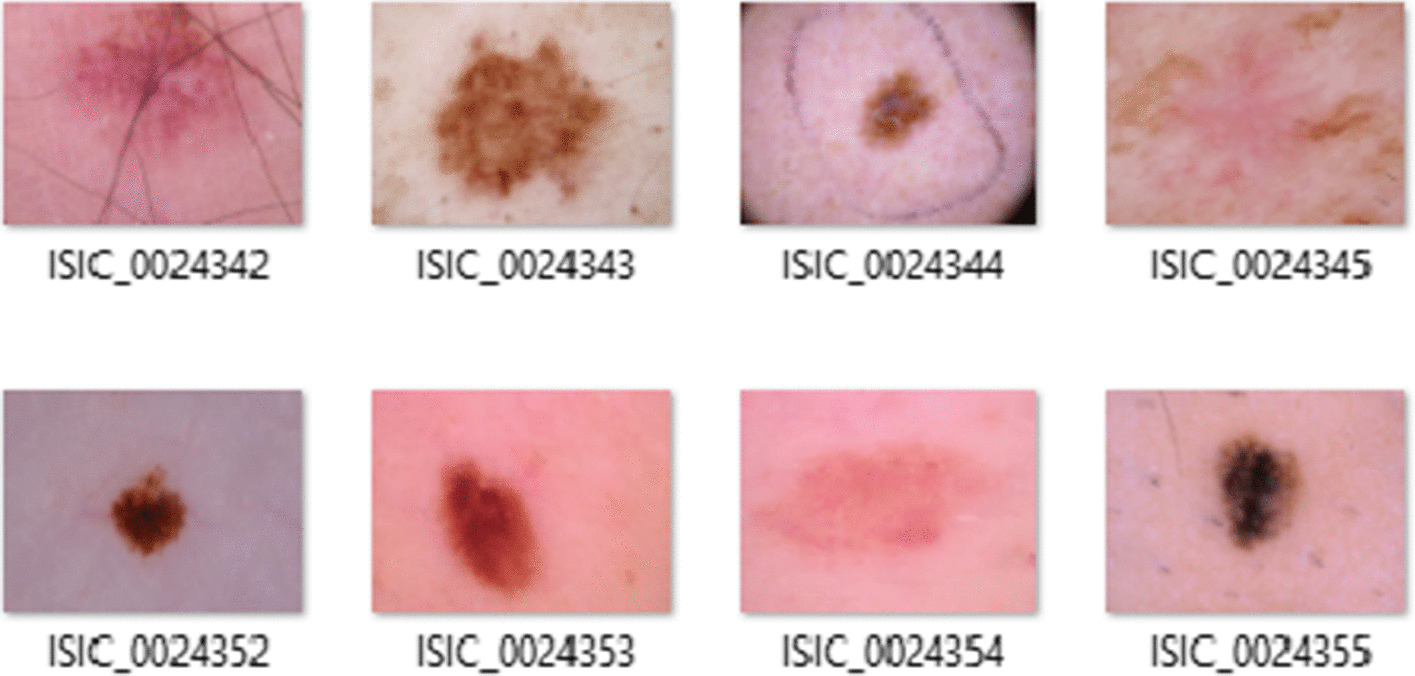
Sample dataset

Table 2 summarizes the findings of this research study. This experimentation is recognized by relating numerous [51] Ensemble classifiers, namely, Bagging and Attribute Selected Classifier by using Auto Color Correlogram Filter, Binary Patterns Pyramid Filter, and Color Layout Filter to bring out the optimal results, as specified in Figs. 1, 2 and 3.

**Table 2:** Performance of classifiers on dataset by auto color correlogram filter

S. No.	Ensemble classifier	Accuracy (%)	Precision	Recall	ROC	PRC
1	Bagging with auto color correlogram filter	83.88	0.85	0.84	0.92	0.90
2	Attribute selected classifier with auto color correlogram filter	82.65	0.82	0.83	0.92	0.72
3	Bagging with binary patterns pyramid filter	85.06	0.84	0.85	0.90	0.88
4	Attribute selected classifier with binary patterns pyramid filter	89.97	0.90	0.90	0.91	0.91
5	Bagging with color layout filter	85.77	0.85	0.86	0.87	0.88
6	Attribute selected classifier with color layout filter	90.96	0.91	0.91	0.95	0.87

Table 2 displays the results of using a few different image enhancing algorithms with a few different classifiers. It has been found that the Attribute Selected Classification algorithm with the implementation of the Auto Color Correlogram Filter achieves an accuracy level of 82.65%, the Bagging algorithm with the use of the Binary Patterns Pyramid Filter of image feature extraction achieves an accuracy level of 85.06%, and the Bagging algorithm with the use of the Auto Color Correlogram Filter of meta category classification achieves an accuracy level of 83.88%.

Table 2 displays the accuracy scores achieved by various classifiers using various image enhancing strategies. Precision levels for the Auto Color Correlogram Filter-based Bagging of meta-category classification algorithms are 0.85 and 0.82, respectively; the Binary Patterns Pyramid Filter-based Bagging of image feature extraction algorithms is 0.84, and the Attribute Selected Classification algorithms is 0.82.

Table 2 shows the classifiers that were chosen alongside the corresponding picture improvement methods. The recall levels of the Attribute Selected Classification and Bagging algorithms are 0.83 and 0.84, respectively, while the recall levels of the Bagging and Binary Patterns Pyramid Filter of image feature extraction are 0.85 and 0.84, respectively.

Table 2 displays the ROC values achieved by the chosen classifiers using the chosen image enhancing methods. The ROC for the Attribute Selected Classification algorithm that uses an Auto Color Correlogram Filter is 0.92, the ROC for the Bagging algorithm that uses an image feature extraction method based on a Binary Patterns Pyramid Filter is 0.90, and the ROC for the Bagging algorithm that uses an Auto Color Correlogram Filter for meta category classification is 0.92.

Table 2 displays the PRC values produced using various image enhancement methods and several classifiers. For example, the PRC level of 0.90 is achieved by the Bagging of meta-category classification algorithms using the Auto Color Correlogram Filter, while the PRC level of 0.72 is achieved by the Attribute Selected Classification algorithms using the Auto Color Correlogram. The PRC level of 0.88 is achieved by the Bagging of image feature extraction algorithms using the Binary Patterns Pyramid Filter.

**Fig. 4:**
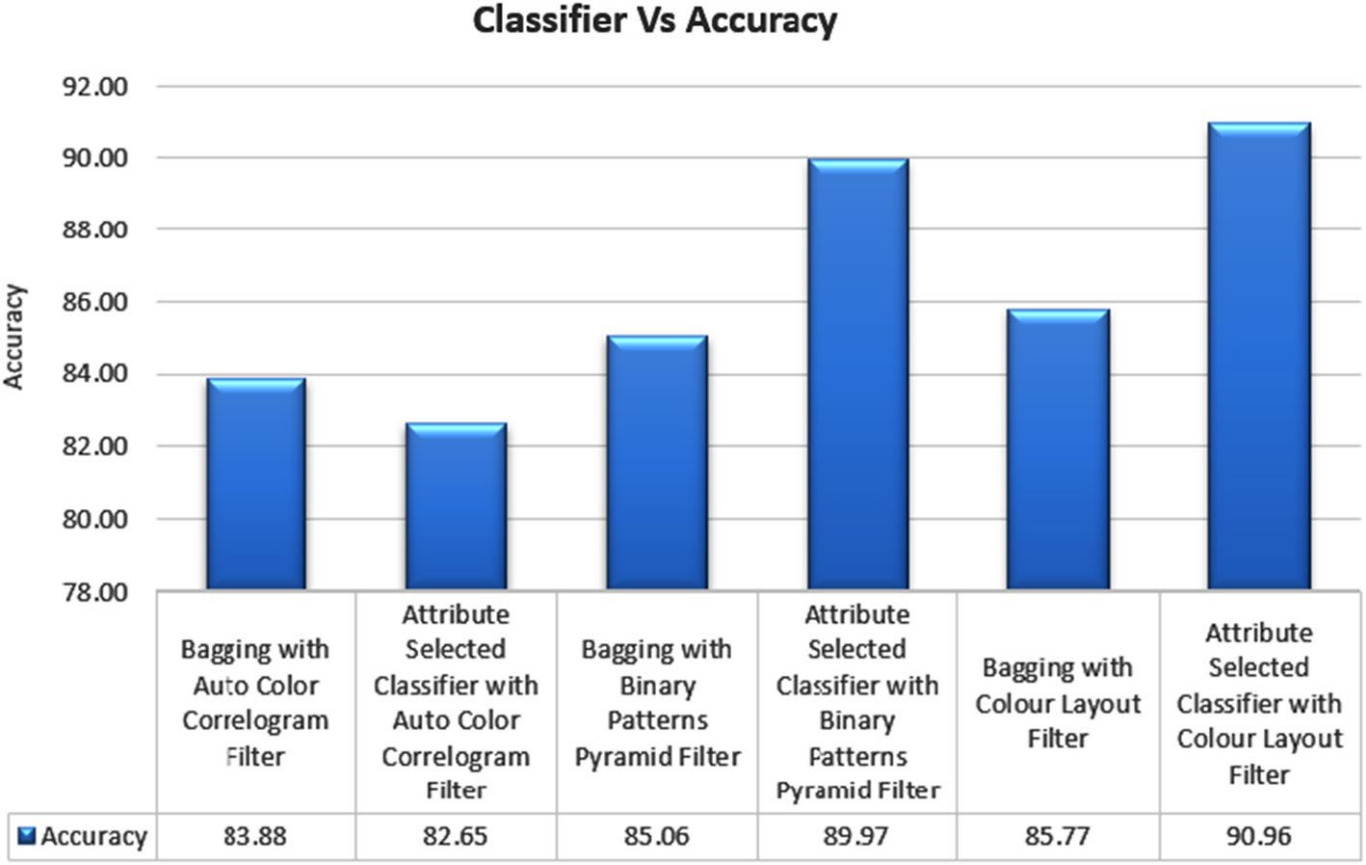
Accuracy performance of classifiers

Figure 4 depicts the results of using the recommended classifiers in conjunction with various picture feature extraction methods in terms of accuracy. This chart compares the accuracy of different classifier ensembles employing different picture filters. The Attribute Selection Classifier algorithm employing the Auto Color Correlogram Filter generates the least accurate value of 82.65 percent. Attribute Selection Classifier with Color Layout filter has the greatest accuracy of 90.96%. The accuracy scores range from 83.88 percent to 89.97 percent for the Bagging with Auto Color Correlogram Filter, Bagging with Binary Patterns Pyramid Filter, Bagging method with Color Layout Filter, and Attribute Selection Classifieralgorithm with Binary Patterns Pyramid Filter.

**Fig. 5:**
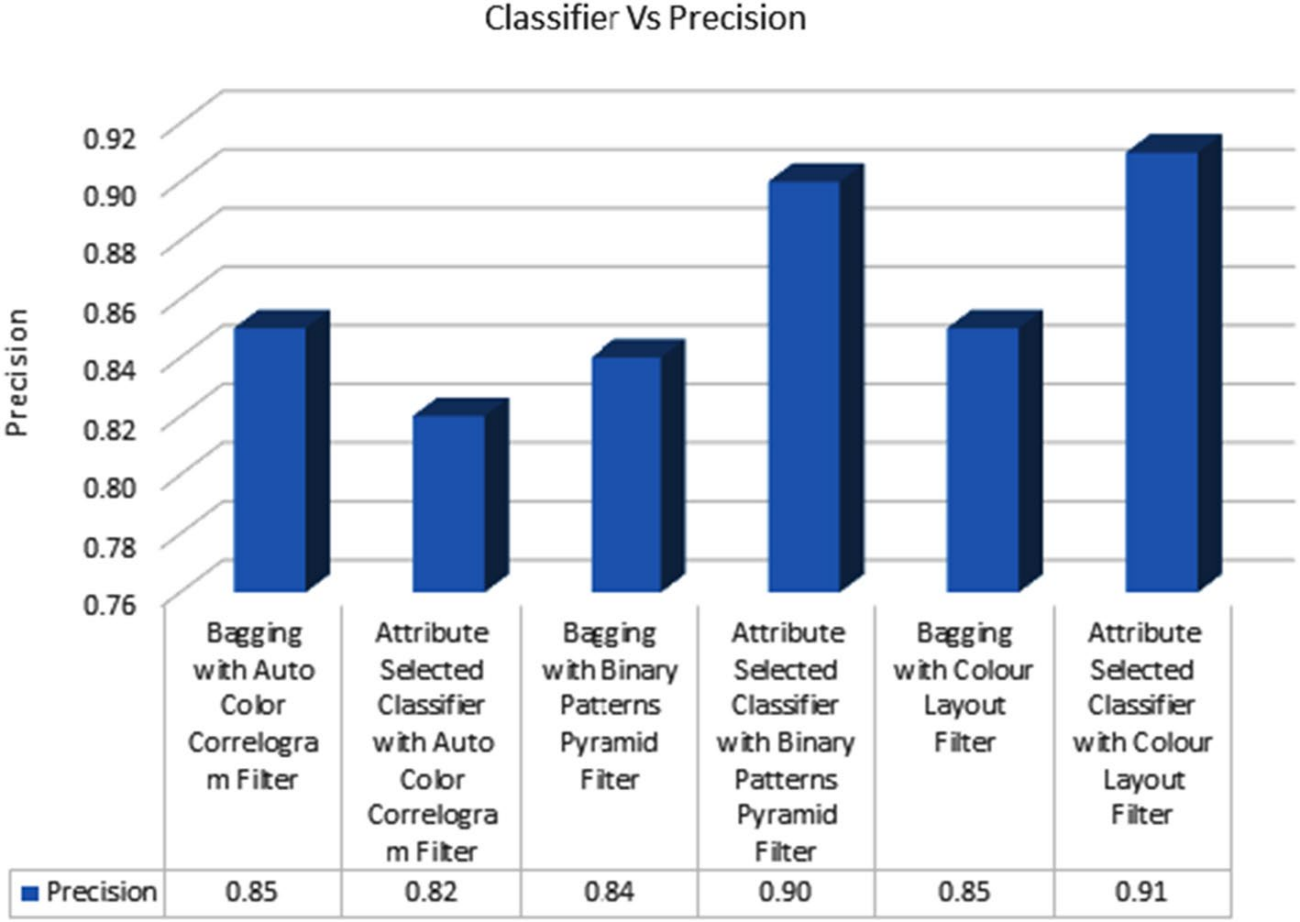
Precision performance of classifiers

Figure 5 displays the accuracy values derived from the chosen classifiers using the chosen picture feature extraction methods. All the groups of classifiers using different image filters are compared in terms of precision here using a graphical representation. The Attribute Selection Classifier algorithm employing the Auto Color Correlogram Filter yields an accuracy of 0.82 at its lowest setting. Attribute Selection Classifier with Color Layout Filter achieves the greatest precision of 0.91. Classifiers like Bagging with Auto Color Correlogram Filter, Bagging with Binary Patters Pyramid Filter, Bagging method utilizing Color Layout Filter, and Attribute Selected Classifier algorithm using Binary Patters PyramidFilter all have precision levels between 0.85 and 0.90.

**Fig. 6:**
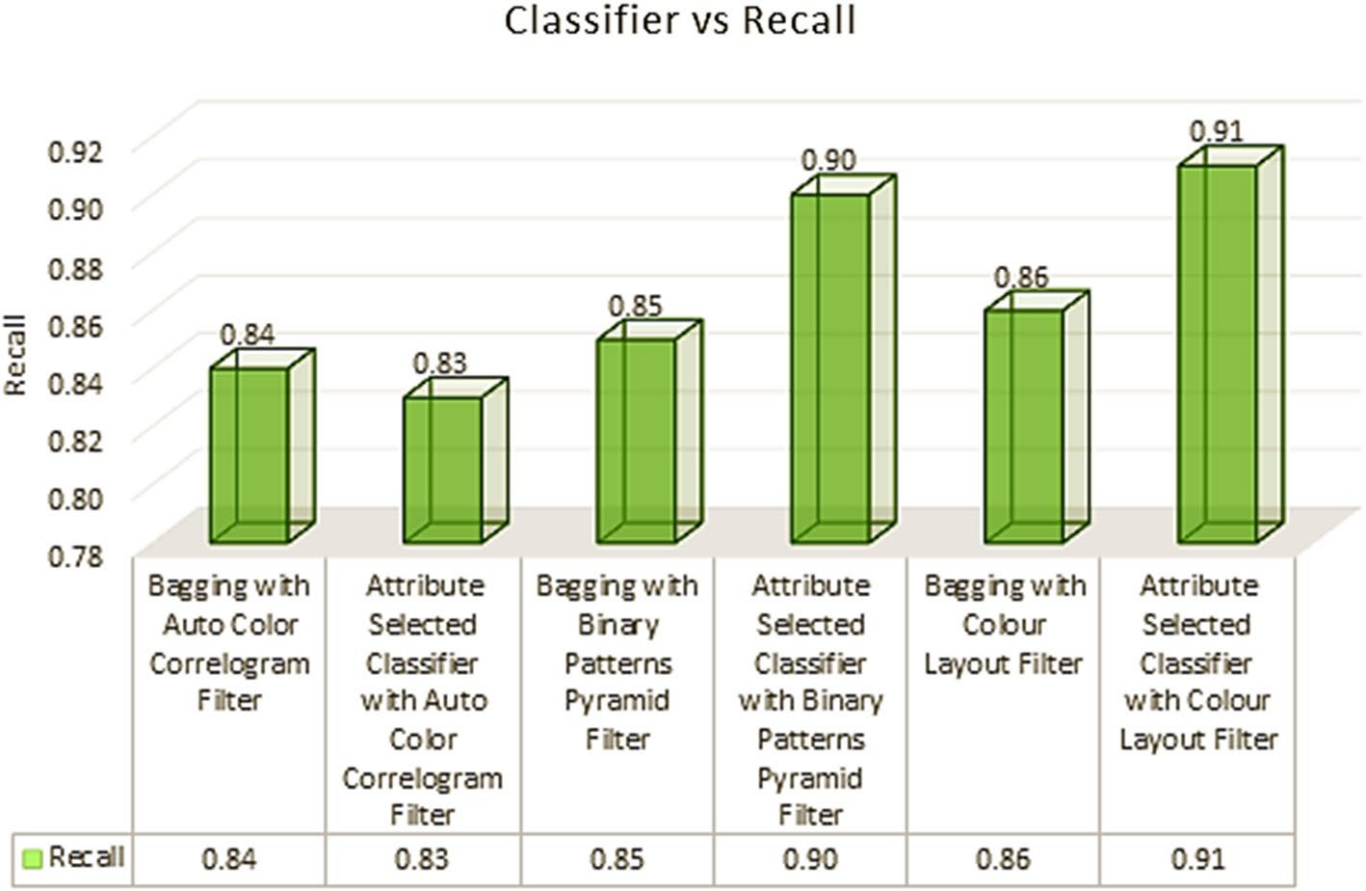
Recall performance of classifiers

The aforementioned chart depicts the classifiers' recall performances after being chosen. Here, we see how different classes of classifiers using different image filters compare with respect to recall rates. With the Auto Color Correlogram Filter, the Attribute Selection Classifier method generates a recall value of 0.83, which is the lowest possible. With an Attribute Selection Classifier using a Color Layout Filter, we achieve a recall of 0.91. Recall values for the remaining classifiers range from 0.84 on the recall scale to 0.90 on the recall scale, and they include The utilization of various bagging algorithms, namely Bagging with Auto Colour Correlogram Filter, Bagging with Binary Patterns Pyramid Filter, Bagging method using Colour Layout Filter, and Attribute Selected Classifier algorithm using Binary Patterns Pyramid Filter, has been investigated. (see Fig. 6).

**Fig. 7:**
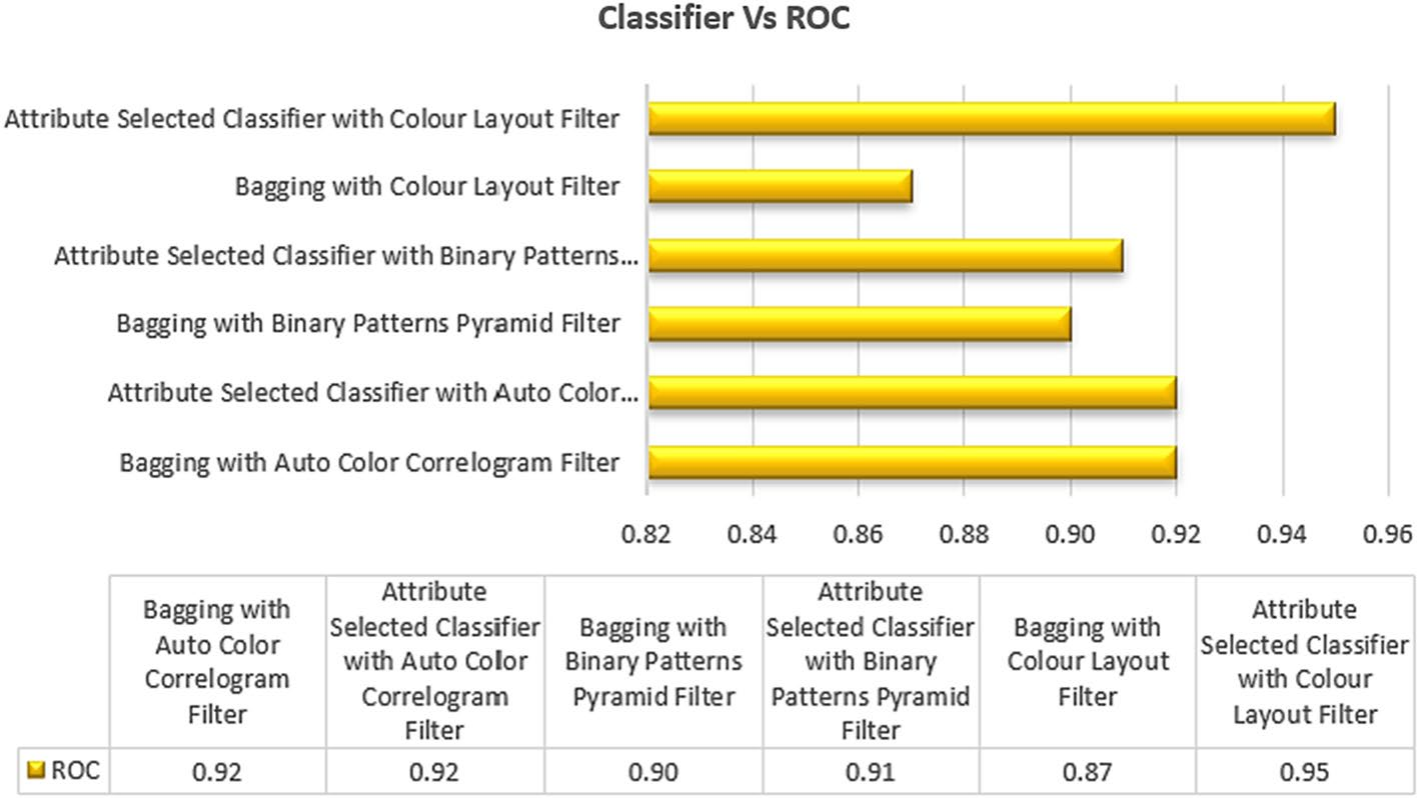
ROC performance of classifiers

Figure 7 displays the ROC values obtained by various classifiers using various image feature extraction strategies. This diagram depicts the comparison of ROC values across all categories of classifiers employing different image filters. With the Color Layout Filter, the Bagging method generates a ROC value of 0.87, which is the lowest possible. The highest ROC value is 0.95, which is having an Attribute Selected Classifier by implementing a Colour Layout Filter.

The ROC values for the other classifiers range from 0.90 to 0.92, and they include the Bagging using the Binary Patters Pyramid Filter and the Attribute Selection Classifier method using the Binary Patterns Pyramid Filter. In this case, the ROC value for using the Auto Color Correlogram Filter for either bagging or crediting the selected classifier is the same.

**Fig. 8:**
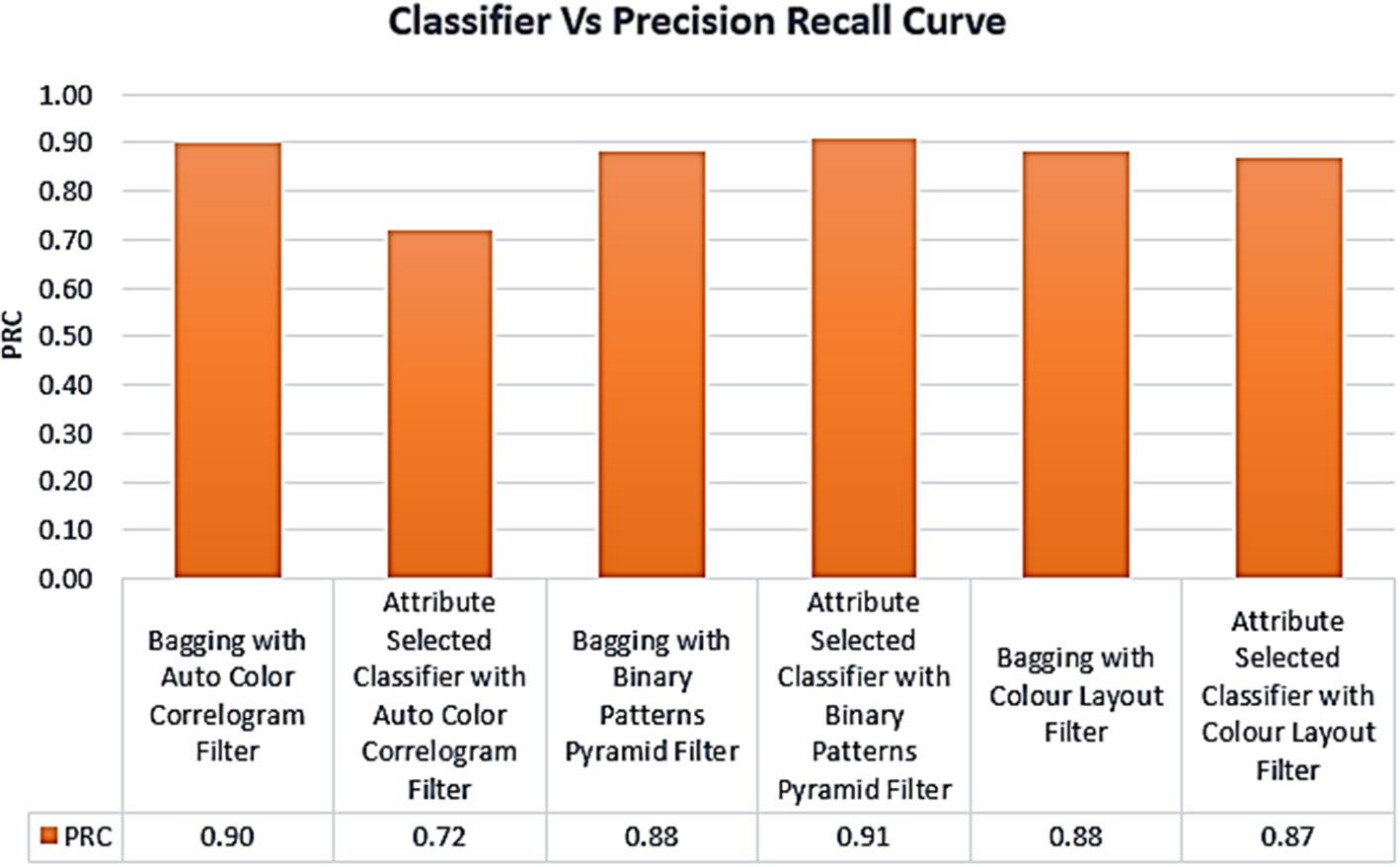
PRC performance of classifiers

Figure 8 displays the PRC values derived using the aforementioned classifiers and feature extraction methods for images. This chart shows the comparison of PRC scores across all categories of classifiers employing different image filters. Attribute Selection Classifier with Auto Color Correlogram Filter generates the lowest PRC value of 0.72. Attribute Selection Classifier using Binary Patterns Pyramid Filter yields the greatest PRC value of 0.91.

PRC values range from 0.87 to 0.90 for the Attribute Chosen Classifier with Colour Layout Filter, Bagging with Binary Patterns Pyramid Filter, Bagging with Colour Layout Filter, and Bagging with Auto Color Correlogram Filter. The PRC value of 0.88 is shared by two models: the Bagging algorithm using the Color Layout Filter and the Bagging method using the Binary Patterns Pyramid Filter.

Table 3 displays the kappa values obtained from the various classifiers using the chosen image enhancement methods. Auto Color Correlogram Filter is used in the Bagging of meta category classification algorithms, yielding a 0.70 kappa statistic value; its implementation in the Attribute Selected Classification algorithms yields a 0.66 kappa statistic value; the Binary Patterns Pyramid Filter is used in the Bagging of image feature extraction algorithms, yielding a 0.71 kappa statistic value; and the Attribute Selected Classification algorithms yield a 0.66 kappa statistic value.

**Table 3:** Kappa, F1 Score, MCC, and performance of classifiers

S. No.	Ensemble classifier	Kappa statistic	F-measure	MCC	Time taken to build model
1	Bagging with auto color correlogram filter	0.70	0.84	0.73	2.80
2	Attribute selected classifier with auto color correlogram filter	0.66	0.80	0.79	4.19
3	Bagging with binary patterns pyramid filter	0.71	0.84	0.74	1.00
4	Attribute selected classifier with binary patterns pyramid filter	0.79	0.90	0.71	17.95
5	Bagging with color layout filter	0.72	0.85	0.74	27.17
6	Attribute selected classifier with color layout filter	0.80	0.91	0.82	16.97

Table 3 displays the F-Measure values obtained from the various classifiers using the aforementioned image enhancing methods. The Attribute Selected Classification algorithm by implementing Auto Color Correlogram has 0.80F-Measure value, while the Bagging algorithm for the meta category by using the Binary Patterns Pyramid Filter of image features extraction achieves 0.84F-Measure value.

Table 3 displays the MCC values obtained using various image enhancing approaches and several classifiers. The MCC for the Attribute Selected Classification algorithm that implements the Auto Color Correlogram Filter is 0.79, and the MCC for the Bagging algorithm that uses the Binary Patterns Pyramid Filter for image feature extraction is 0.74.

Table 3 displays the time it took to construct their models using the chosen classifiers and the chosen picture enhancing methods. It took 2.80 s for the Bagging of meta category classification algorithm to build its model using Auto Color Correlogram Filter, and it took 4.19 s for the Attribute Selected Classification algorithm to build its model using the Binary Patterns Pyramid Filter image feature extraction technique. In that time, it has accumulated 1 s of data and built a model. By using the Binary Patterns Pyramid Filter method of extracting features from images, the Attribute Selection Classification algorithm can reliably classify images based on their attributes. Building a model with the Color Layout Filter of Image enhancement approach Filter took 27.17 s with the Bagging classifier, and 16.97 s with the Attribute Selection Classification algorithm.

**Fig. 9:**
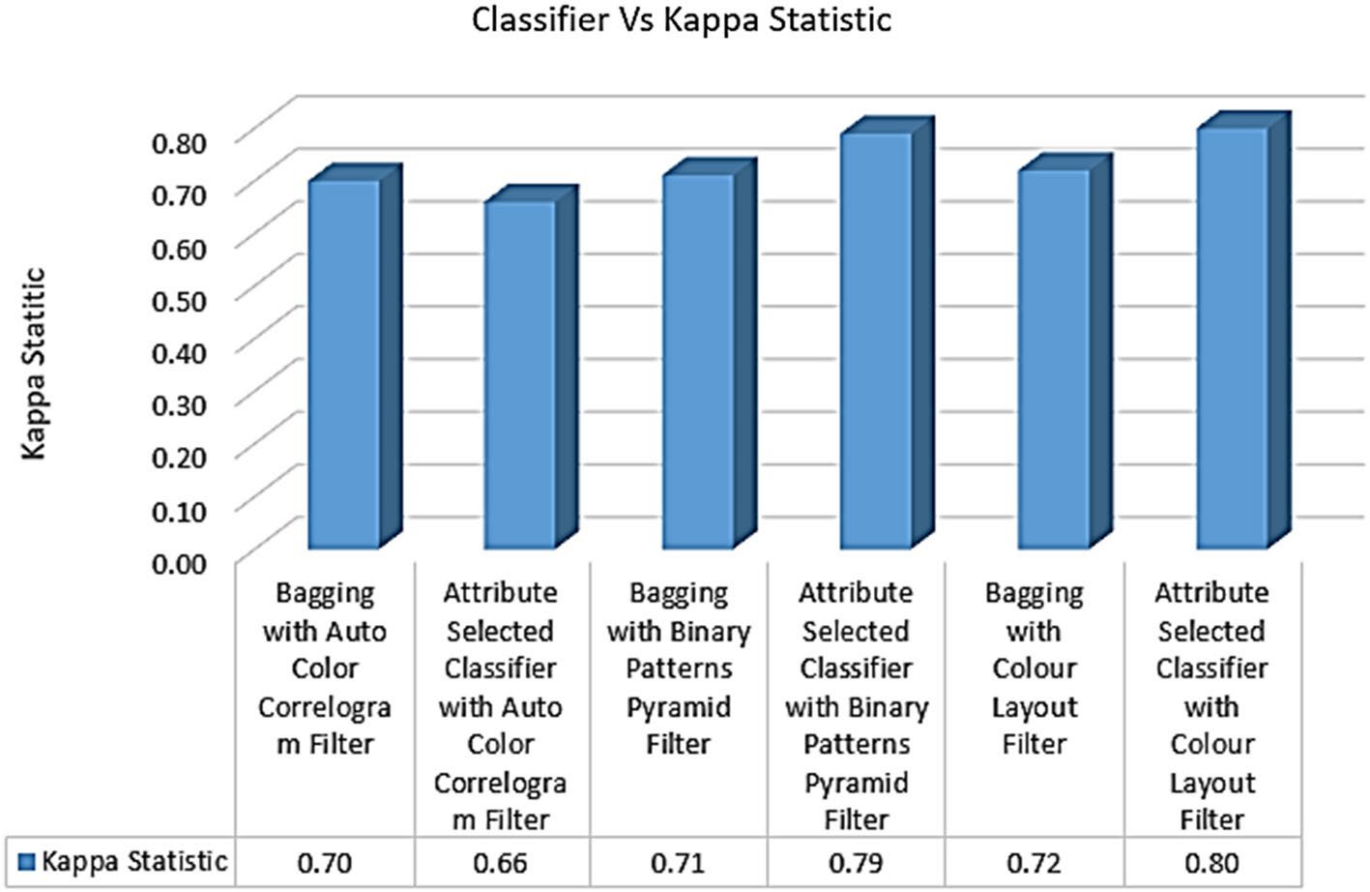
Kappa performance of classifiers

Figure 9 displays the Kappa values attained by various classifiers employing various picture feature extraction methods. Kappa values are evaluated for every classifier group using each image filter, and the results are plotted here. Attribute Selection Classifier with Auto Color Correlogram Filter yields the lowest kappa value of 0.66. The Attribute Selected Classifier with the Color Layout Filter yielded the highest kappa statistic value (0.80).

There is a wide range of kappa values between 0.70 and 0.79 for the four different bagging methods (Bagging with Auto Color Correlogram Filter, Bagging with Binary Patterns Pyramid Filter, Bagging with Color Layout Filter, and Attribute Selection Classifier with Binary Patterns Pyramid Filter).

**Fig. 10:**
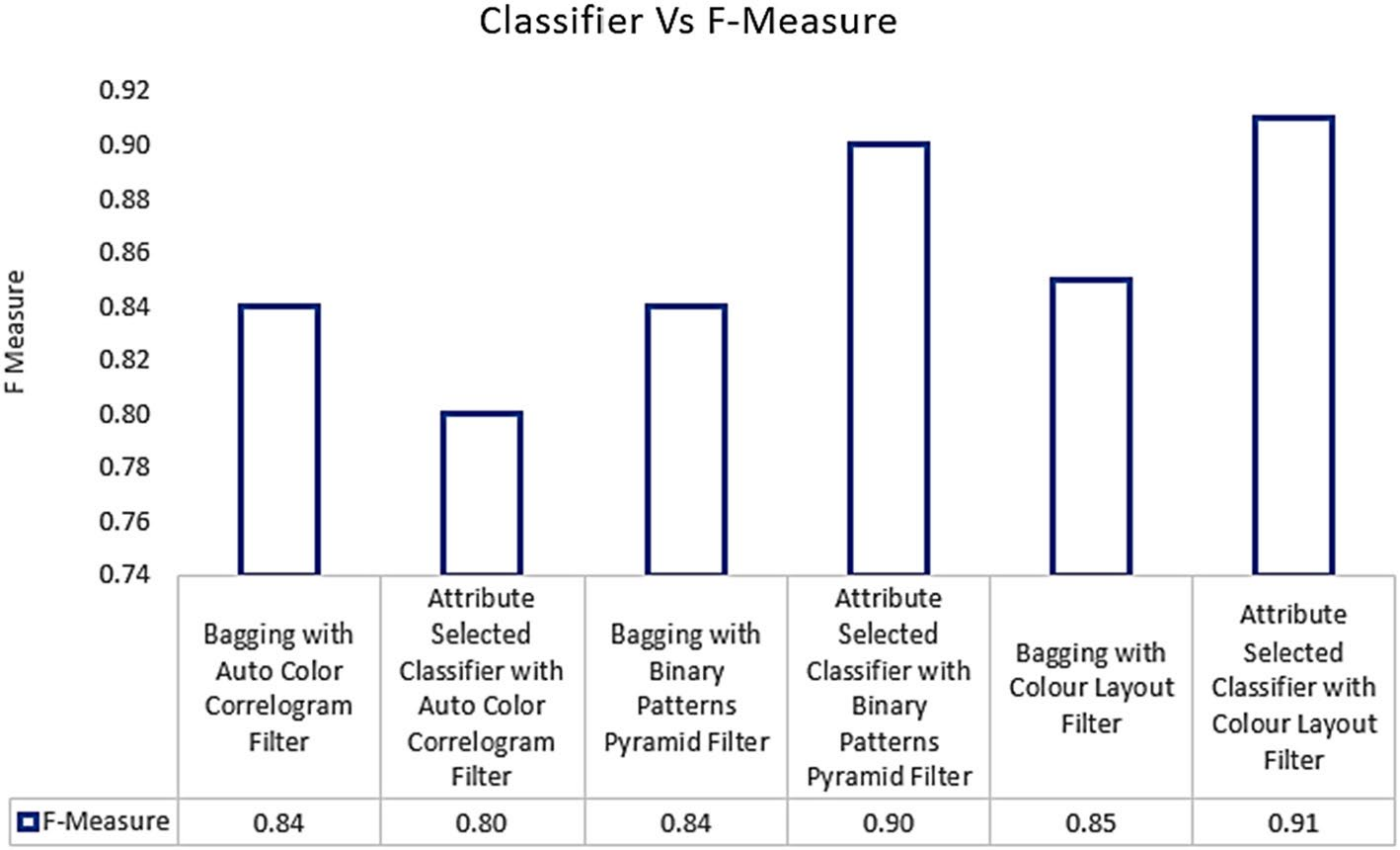
F-Measure performance of classifiers

The F-Measure values obtained from the nominated classifiers with selected image feature extraction techniques are shown in Fig. 10. This graph characterizes the contrast of F-Measure values for all the groups of the classifiers with various image filters. The least F-Measure value is 0.80, which is created by the Attribute Selected Classifier with Auto Color Correlogram Filter. The highest F-Measure value is 0.91, which is having an Attribute Selected Classifier by implementing Color Layout Filter.

The F-Measure values of the Bagging with Auto Color Correlogram Filter, Bagging with Binary Patterns Pyramid Filter, Bagging with Color Layout Filter, and Attribute Selected Classifier with Binary Patterns Pyramid Filter range from 0.84 to 0.90. Hence, bagging using Auto Color Correlogram Filter and bagging using Binary Patterns Pyramid Filter models have the same F-Measure value, which is 0.84 of the F-Measure value.

**Fig. 11:**
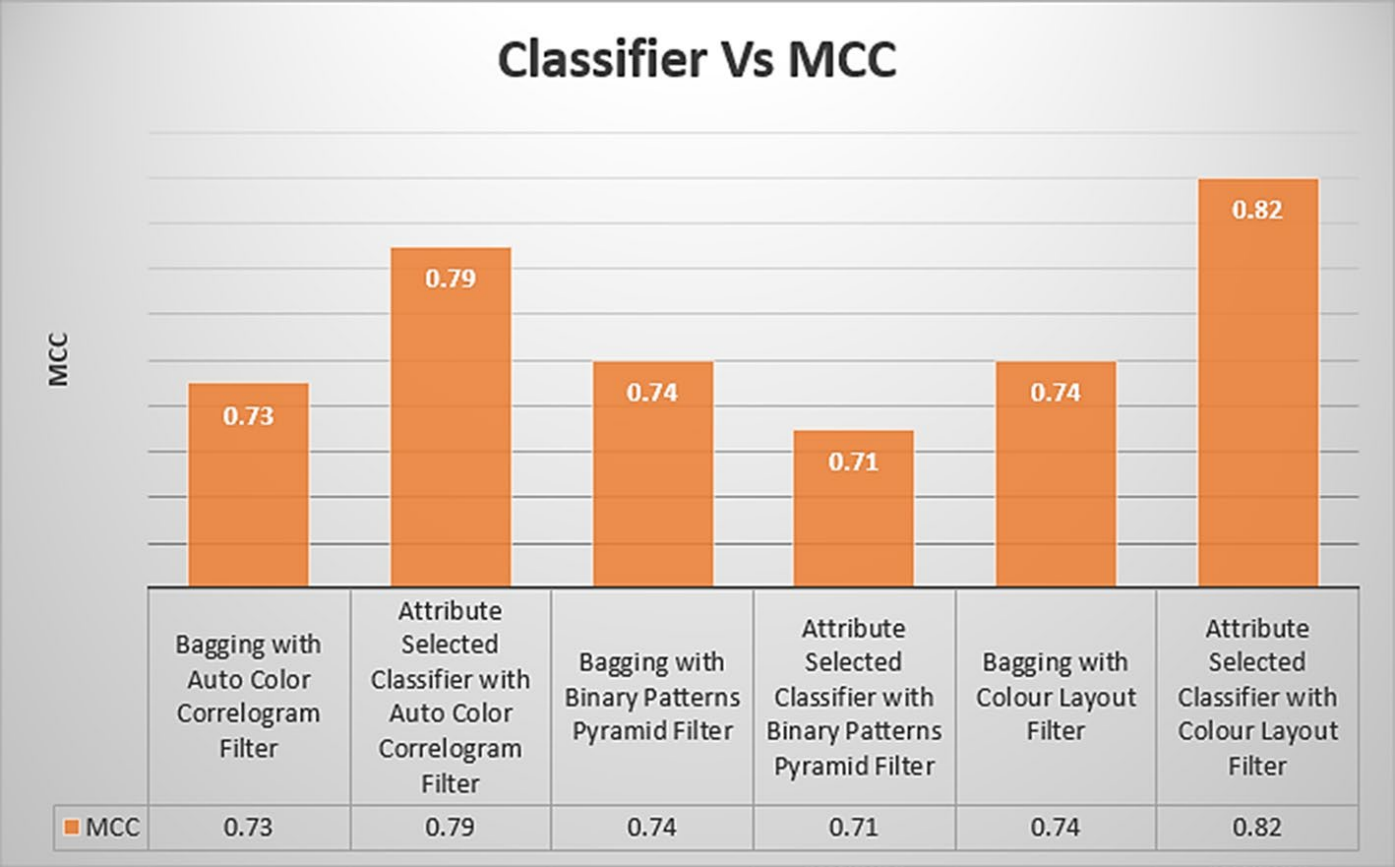
MCC performance of classifiers

The MCC values obtained from the particular classifiers with selected image feature extraction techniques are shown in Fig. 11. This graph represents the assessments of MCC values for all the types of classifiers with various image filters. The least MCC value is 0.71, which is produced by the Attribute Selected Classifier with Binary Pattern Pyramid Filter. The highest MCC value is 0.82, which has an Attribute Selected Classifier by implementing Color Layout Filter.

The Bagging with Auto Color Correlogram Filter, Bagging with Binary Patterns Pyramid Filter, Bagging with Color Layout Filter, and Attribute Selected Classifier with Auto Color Correlogram have MCC values from 0.73 of the MCC value to 0.79 of the MCC value. As a result, bagging with the Color Layout Filter model and bagging with the Binary Patterns Pyramid Filter model have the same MCC value, which is 0.74 of the MCC value.

**Fig. 12:**
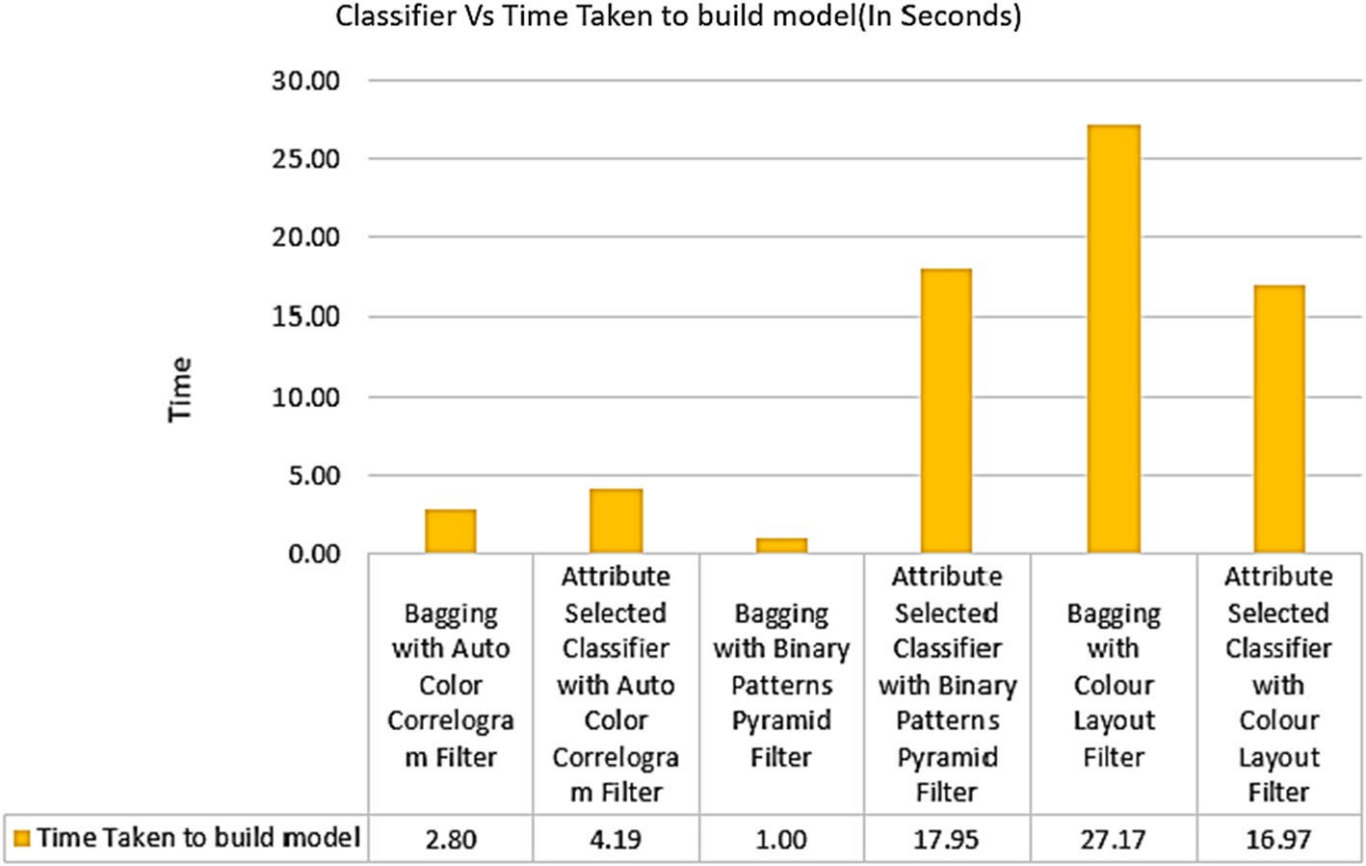
Time- consumption performance of classifiers

The time consumption to build their models acquired from the particular classifiers with selected image feature extraction techniques is shown in Fig. 12. This graph exemplifies the contrasts in time consumption for building models for all the categories of the classifiers with various image filters. The least time-consuming way to build a model is 1 s, which is produced by the Attribute Selected Classifier with Auto Color Correlogram Filter. The highest time consumption is 27.17 s to build a model which has Bagging by implementing Color Layout Filter.

Bagging with Auto Color Correlogram Filter, Attribute Selected Classifier with Color Layout Filter, and Attribute Selected Classifier with Binary Patterns Pyramid Filter all take between 2.80 and 17.95 s.

Table 4 displays the Mean Absolute Errors achieved by several classifiers using various image improvement strategies. Mean absolute error values for meta-category-classification-algorithm-Bagging with the Auto Color Correlogram Filter are 0.23 and 0.38, respectively; for image-feature-extraction-algorithm-Bagging with the Binary Patterns Pyramid Filter, the values are 0.24 and 0.24, respectively; and for attribute-selected-classification-algorithm-Bagging, they are 0.24 and 0.38, respectively.

**Table 4:** Deviation performance of classifiers

S. No.	Ensemble classifier	Mean absolute error	Root mean squared error	Relative absolute error (%)	Root relative squared error (%)
1	Bagging with auto color correlogram filter	0.23	0.42	50.66	90.30
2	Attribute selected classifier with auto color correlogram filter	0.38	0.46	96.98	99.73
3	Bagging with binary patterns pyramid filter	0.24	0.40	55.05	82.96
4	Attribute selected classifier with binary patterns pyramid filter	0.25	0.37	54.51	76.18
5	Bagging with colour layout filter	0.27	0.41	62.25	86.34
6	Attribute selected classifier with colour layout filter	0.24	0.30	53.73	74.40

Table 4 displays the Root Mean Squared Error scores achieved by several classifiers using various image enhancing strategies. Using the Auto Color Correlogram Filter in a meta-category classification algorithm yields an RMS error of 0.42, while doing the same with the Attribute Selected Classification algorithm yields an RMS error of 0.46, while using the Binary Patterns Pyramid Filter in an image feature extraction technique yields an RMS error of 0.40.

Table 4 displays the relative absolute errors achieved by several classifiers employing various picture improvement strategies. It has been found that the Attribute Selected Classification algorithm, when using Auto Color Correlogram, has a relative absolute error value of 96.98%, while the Bagging algorithm, when using the Binary Patterns Pyramid Filter of the image feature extraction technique, has a relative absolute error value of 55.05%.

Table 4 displays the Root Mean Square Errors achieved by several classifiers using various picture enhancing methods. The root relative squared value is 90.30% for the Bagging of the meta category classification algorithm using the Auto Color Correlogram Filter, and it is 99.73% for the Attribute Selected Classification algorithm using the same filter. The Bagging of the image feature extraction technique using the Binary Patterns Pyramid Filter yields an even lower value of 82.96% for the root relative squared value.

**Fig. 13:**
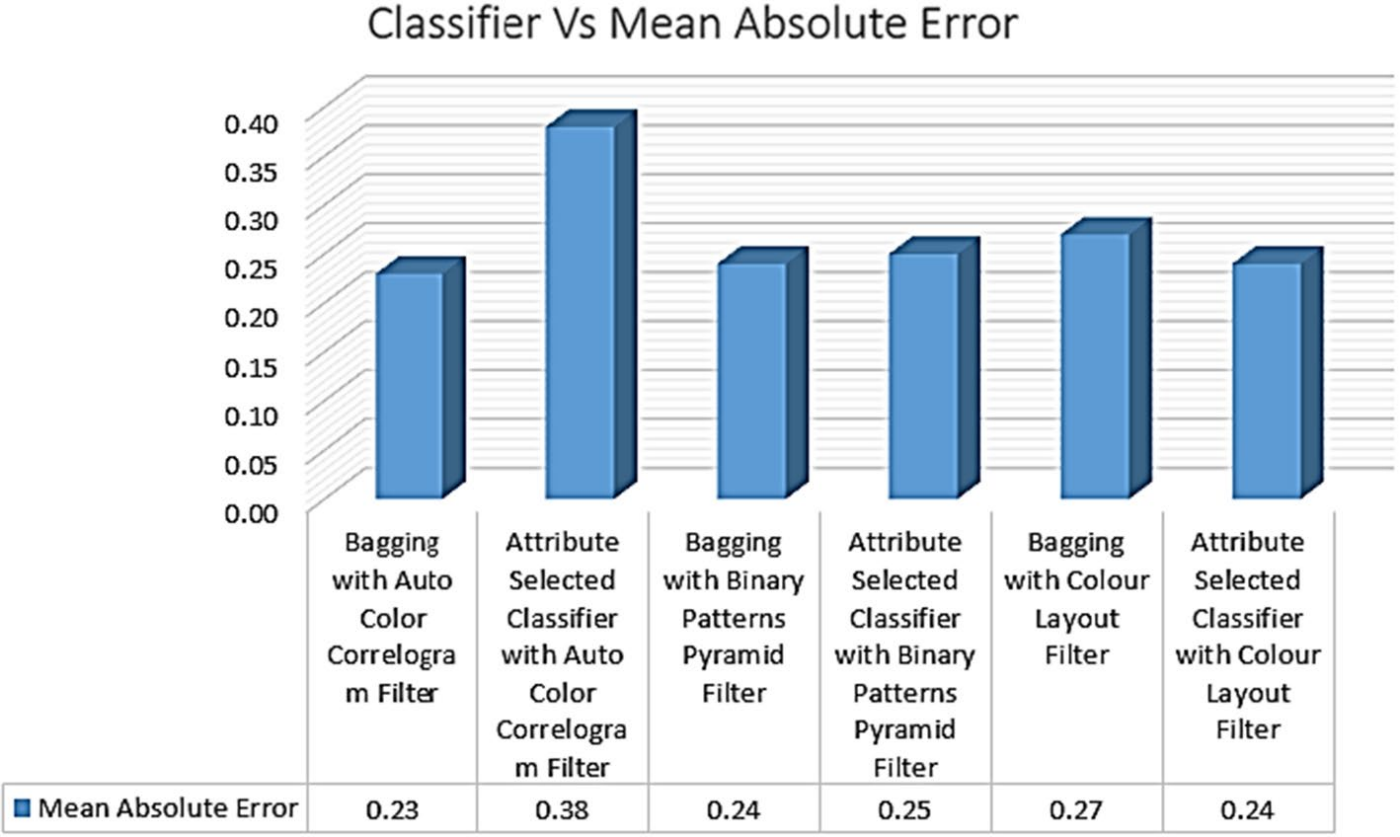
MAE performance of classifiers

The Mean Absolute Error values obtained from the selected classifiers with selected image feature extraction techniques are shown in Fig. 13. This graph represents the comparisons of MAE values for all the categories of the classifiers with various image filters. The least mean absolute error value is 0.23, which is produced by bagging with the Auto Color Correlogram Filter. The highest MAE value is 0.38, which is having an Attribute Selected Classifier by implementing the Auto Color Correlogram Filter. The Bagging with Binary Patterns Pyramid Filter, Attribute Selected Classifier with color Layout Filter, Attribute Selected Classifier with Binary Patterns Pyramid Filter, and Bagging with color Layout Filter have MAE values ranging from 0.24 of mean absolute value to 0.27 of MAE value. Hence, bagging using Binary Patterns Pyramid Filter and bagging using Attribute Selected Classifier with color Layout Filter models have the same MAE value, which is 0.24 of the MAE value.

**Fig. 14:**
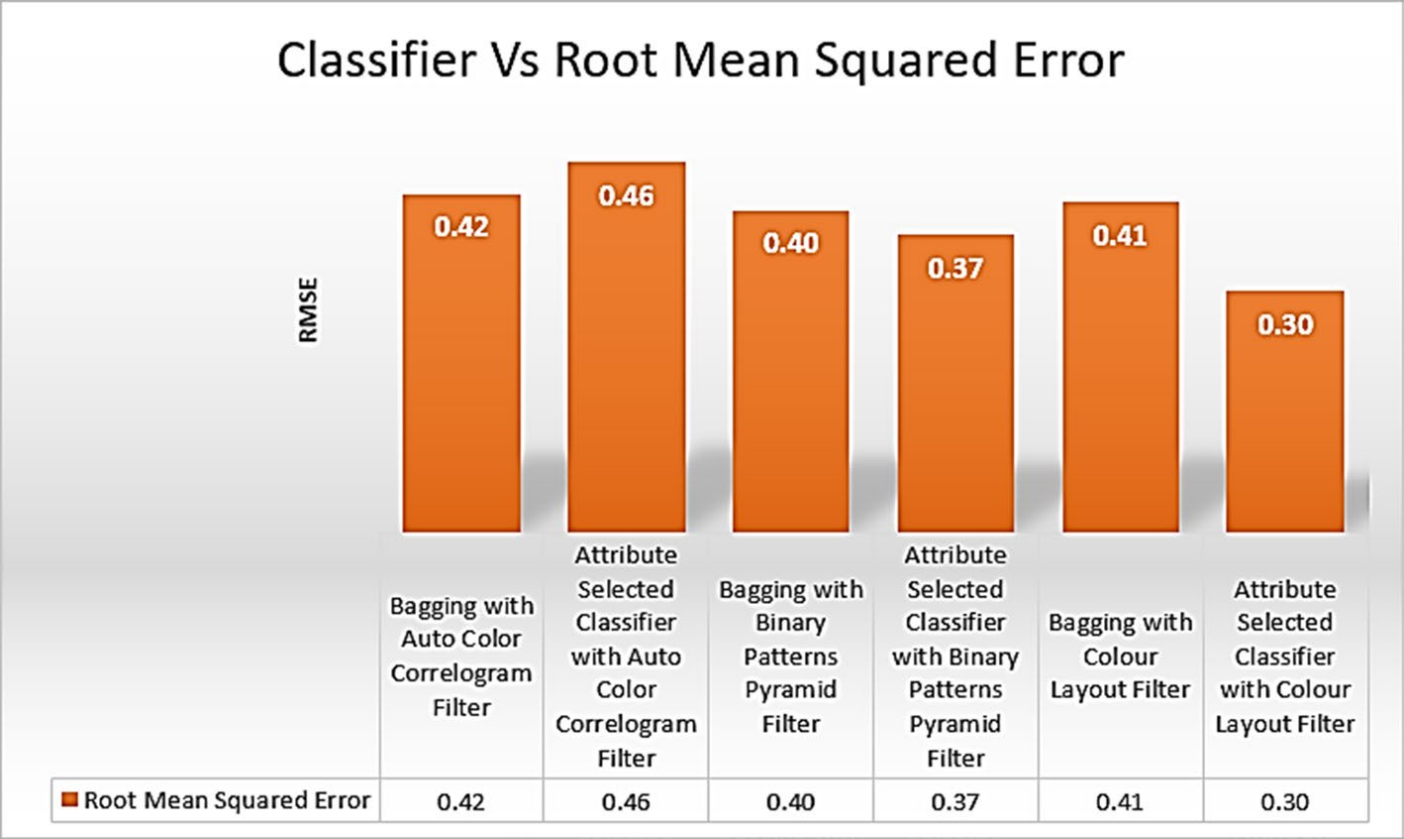
RMSE performance of classifiers

The Root Mean Squared Error values obtained from the selected classifiers with selected image feature extraction techniques are shown in Fig. 14. It represents the comparisons of RMSE values for all the categories of the classifiers with various image filters. The least RMSE value is 0.30, which is produced by the Attribute Selected Classifier with color Layout Filter. The highest RMSE value is 0.46, which is having an Attribute Selected Classifier by implementing Auto Color Correlogram Filter. The Attribute Selected Classifier with Binary Patterns Pyramid Filter, Bagging with Binary Patterns Pyramid Filter, Bagging with color Layout Filter, and Bagging with Auto Color Correlogram Filter has a RMSE of 0.37 of RMSE to 0.42 of RMSE.

**Fig. 15:**
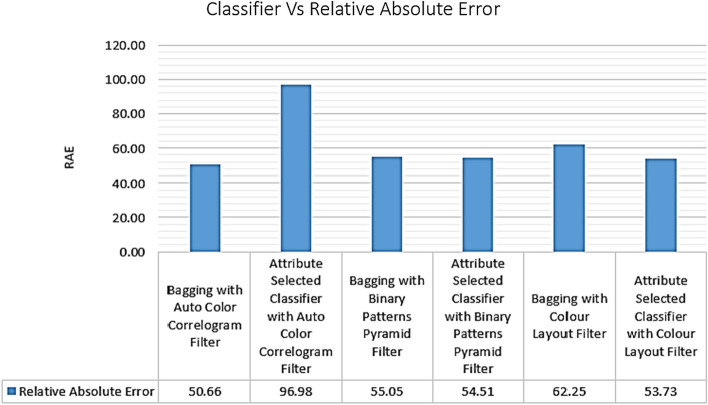
RAE performance of classifiers

The relative absolute error values obtained from the selected classifiers with selected image feature extraction techniques are shown in Fig. 15. This graph represents the comparisons of RAE values for all the categories of the classifiers with various image filters. The least RAE value is 50.66% of the RAE value, which is produced by Bagging with Auto Color Correlogram Filter. The highest RAE value is 96.98% of the RAE value, which is having an Attribute Selected Classifier by implementing Auto Color Correlogram Filter.

The Attribute Selected Classifier with color Layout Filter, Attribute Selected Classifier with Binary Patterns Pyramid Filter, Bagging with Binary Patterns Pyramid Filter, and Bagging with color Layout Filter have RAE values from 53.73% of RAE value to 62.25% of RAE value.

**Fig. 16:**
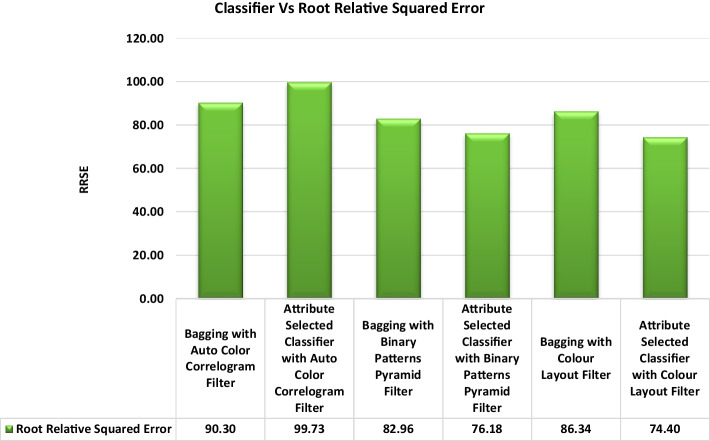
RRSE performance of classifiers

The Root Relative Squared Error values obtained from the selected classifiers with selected image feature extraction techniques are shown in Fig. 16. It represents the comparisons of RRSE values for all the categories of the classifiers with various image filters. The least RRSE value is 74.40% of RRSE, which is produced by the Attribute Selected Classifier with Color Layout Filter. The highest root relative squared error is 99.73% of the RRSE value, which is having an Attribute Selected Classifier by implementing Auto Color Correlogram Filter.

The Attribute Selected Classifier with Binary Patterns Pyramid Filter, Bagging with Binary Patterns Pyramid Filter, Bagging with Color Layout Filter and Bagging with Auto Color Correlogram Filter are RRSE values from 76.18% of RRSE value to 90.30% of RRSE value.

## Discussions

The previous research [60] took into account three separate datasets, including MED-NODE, Dermatology Information System & DermQuest, and the ISIC 2017. They say that 85% of the datasets were used for training and fine-tuning the proposed DCNN, while the remaining 15% were used for testing and verification. The classification accuracy for MED-NODE (99.29%), DermIS & DermQuest (98.15%), and ISIC 2017 (98.14%) all increased dramatically thanks to this strategy when using modified GoogleNet. Yet, our suggested system dictates that the 10% of the ISIC 2018 revised dataset. As a result, an ensemble was generated using the ACCF, BPPF, and CLF approaches, with ASC's CLF model demonstrating 95% accuracy.

## Conclusion

The present study demonstrates that the Attribute Selected Classifier, belonging to the ensemble category, while utilizing the color Layout Filter model, yields a very effective output with a reduced number of error values. The accuracy, precision, recall, ROC, PRC, kappa statistic, F-Measure, and MCC values are 90.96%, 0.91, 0.94, 0.81, and 0.82, respectively. These values were obtained using the Attribute Selected Classifier with the implementation of the Colour Layout Filter. Skin cancer is the most commonly occurring and hazardous type of cancer in the human population. Melanoma is a type of skin cancer that has the potential to be life-threatening. Early detection greatly increases the likelihood of successful treatment and cure. The biopsy process is the established method for diagnosing melanoma. The aforementioned procedure can be characterized by its time-intensive nature and the potential for inducing discomfort. The present study presents a computer-aided detection approach for the early identification of melanoma. This study presents a diagnostic system that utilizes the Attribute Selected Classifier of Ensemble Category with the color Layout Filter model methodologies to achieve effective results. The image of the affected skin undergoes a series of preprocessing procedures before being enhanced and refined.

## Data Availability

The datasets used during the current study are available from the corresponding author on reasonable request.
